# A Decade in Hijacked Journals: What Will be the Future Trend?

**DOI:** 10.34172/apb.44002

**Published:** 2024-12-05

**Authors:** Mihály Hegedűs, Mehdi Dadkhah, Lóránt Dénes Dávid

**Affiliations:** ^1^Department of Finance and Accounting, Tomori Pál College, Budapest, Hungary.; ^2^Chamber of Hungarian Auditors, Budapest, Hungary.; ^3^Department of Sustainable Tourism, Institute of Rural Development and Sustainable Economy, Hungarian University of Agriculture and Life Sciences (MATE), Gödöllő, Hungary; ^4^Department of Tourism and Hospitality, Faculty of Economics and Business John von Neumann University, Kecskemét, Hungary.; ^5^Department of Sustainable Tourism, Institute of Rural Development and Sustainable Economy, Hungarian University of Agriculture and Life Sciences (MATE), Gödöllő, Hungary.; ^6^Savaria Department of Business Economics, Savaria University Centre, Faculty of Social Sciences, Eötvös Loránd University, Szombathely, Hungary.; ^7^Department of Tourism and Hospitality, Kautz Gyula Faculty of Business and Economics, Széchenyi István University, 9026 Győr, Hungary.

**Keywords:** Hijacked journals, Medicine, Bibliometrics, Publication ethics, Artificial intelligence, Sustainable development goals, Circular economy, Circular society

## Abstract

**Purpose:**

Hijacked journals are fraudulent websites that mimic legitimate journals and, by charging authors, publish manuscripts. The current editorial endeavors to provide a close view of current literature. This editorial piece analyzes 10 years of research on hijacked journals and endeavors to shed light on future trends.

**Methods:**

Current research uses a bibliometric approach to analyze data and discuss results. The OpenAlex has been used for data collection. Some of the data analysis was conducted using OpenAlex. The other study was done using Bibliometrix, and the date is limited to publication between 2014 and 2024.

**Results:**

The findings provide a close view of the published literature in terms of access type, growth, topics, most frequent words, country contribution, top publishers, and alignment of literature with sustainable development goals.

**Conclusion:**

The gap in current literature is the limitation in easily usable methods to be accessible by all researchers for hijacked journal detection and data analysis. The use of artificial intelligence can be promising.

## Introduction

 There is literature about hijacked journals, and this phenomenon has been discussed in various papers. The term “hijacked journals” describes fraudulent activity in which a second illegal web domain(s) (or URL) mimics the original website of a legitimate journal to deceive researchers. In this scam, hackers promote the illegal web address as the original websites of journals, then by charging authors, publish manuscripts without peer review.^1,2^ The history of hijacked journals comes back to about 2011, when a cybercriminal registered an expired domain “sciencerecord.com” and launched three hijacked journals “Science Series Data Report”, “Innova Ciencia”, and “Science and Nature” in 2012 after another hijacked journal “Archives des sciences” that was online in 2011.^3^ From that date till now, there have been many cases of journal hijacking, from simple to advanced ones. There are hijacked journals by just launching a similar web domain to original journals or more advanced ones that use the old domain of indexed journals. In some advanced types of journal hijacking, the content can be indexed in Scopus.^1^ In 2024, we published some editorials on this topic to increase awareness of potential audiences in *Advanced Pharmaceutical Bulletin.*^4,5^ However, it seems that the phenomenon of journal hijacking will not be stopped. Conversely, hijacked journals are becoming increasingly extensive and complex. This editorial piece analyzes 10 years of research on hijacked journals and endeavors to shed light on future trends.

## Methods

 Current research uses a bibliometric approach to analyze data and discuss results. Based on Van Leeuwen, “Bibliometrics is the field of science that deals with the development and application of quantitative measures and indicators for sciences and technology based on bibliographic information”.^6^ The OpenAlex (https://openalex.org) has been used for data collection. OpenAlex is a comprehensive database that provides extensive access to literature and allows for a systematic review of literature.^7^ It also provides options for some primary analysis of the provided data. Many studies used OpenAlex to collect related data.^8-10^ Some of the analysis of the data was conducted by using OpenAlex. other part of study has been done by using Bibliometrix, a tool in the R programming language for bibliometric analysis.^11^ The term “hijacked journal*” has been searched in OpenAlex, and the date is limited to publication between 2014 and 2024. OpenAlex provided more results than other citation bases when searching for publications on the topic of hijacked journals. Because results contain papers that include the keyword “hijacked journal” in any sections of the paper, not only the title, abstract, and keywords.

## Result and Discussion

**Figure 1:**
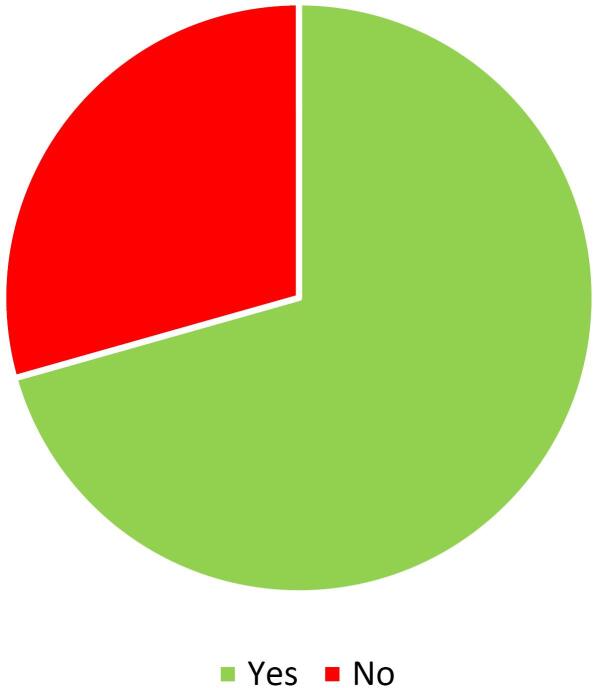
Number of open-access papers

**Figure 2:**
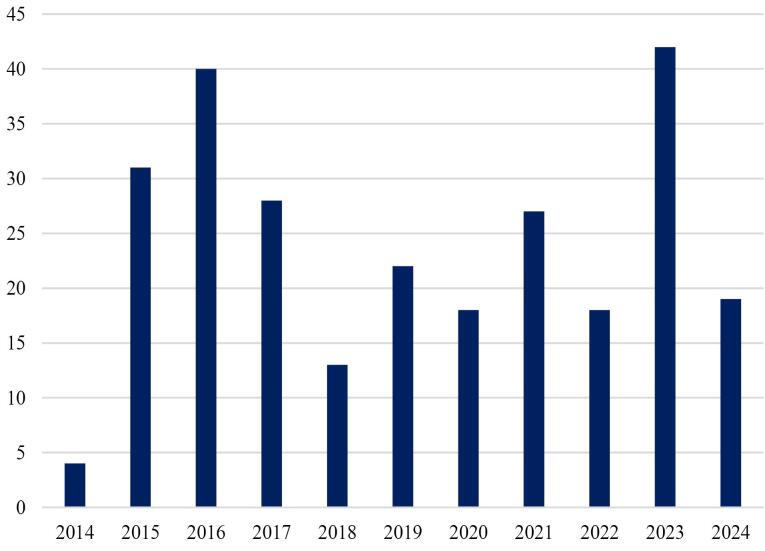
Published papers per year

 The analysis of access types to full texts indicates that nearly 70 percent of publications are open-access (Figure 1). It means that hijacked journals are more concerned with open-access journals than subscription ones. It may be due to hijacked journals usually mimicking open-access journals. Free access to papers on the topic of hijacked journals and their detections can be valuable because it allows the free sharing of related literature and eliminates the number of victims. The number of papers is variants in each year, but there was an increase in 2015 that highlights a concern about the challenges of hijacked journals (Figure 2).

**Figure 3:**
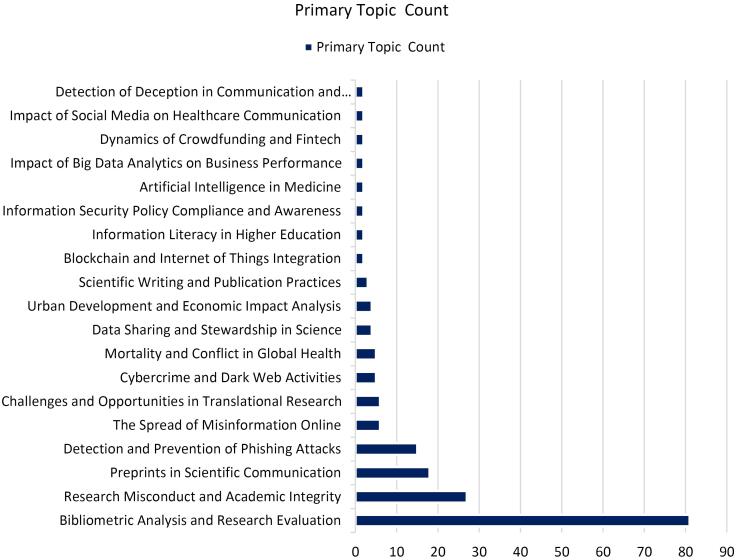
Primary topics in published papers

**Figure 4:**
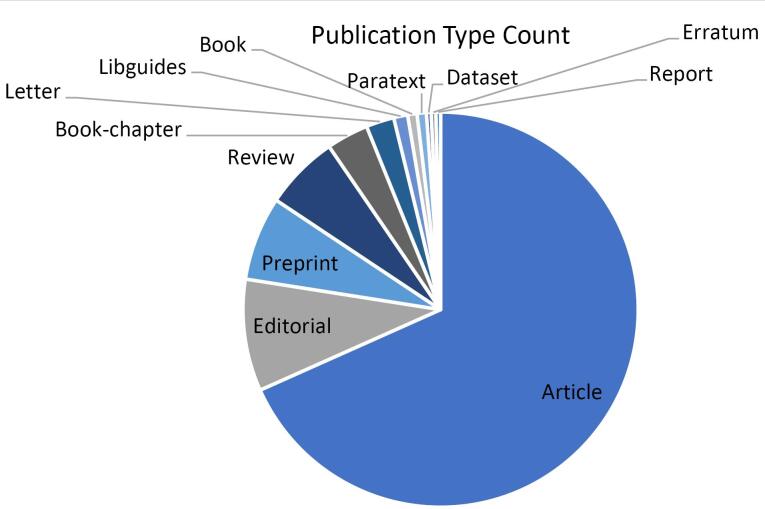
Publication Type

 The primary topics of most papers are bibliometrics, literature reviews, academic misconduct, or phishing attacks (Figure 3). The papers mentioned hijacked journals as the source of unreliable data and tried to warn about such papers to exclude from citation or provide guidelines for authors to prevent from publishing in these journals.^12-14^ These publications endeavor to increase awareness about the bad practices of hijacked journals by introducing this phenomenon, the consequences of publishing in these journals, providing methods for detection of them, etc. The naming of hijacked journals as phishing attacks is another main theme. Both phishing attacks and hijacked journals use identity theft and mimic the original legitimate domain.^15^ The concept of hijacked journals has technical, ethical, and criminal aspects. Academic misconduct, flaws in website structures, and criminal activity shape a hijacking attack. This concept has potential for future research, as current inspection shows that most of the current literature is classified as an article (Figure 4). It means that the hijacked journals’ literature requires more than just providing public awareness, and needs research on providing solutions, evaluating current hurt, considering information security aspects, etc.

**Figure 5:**
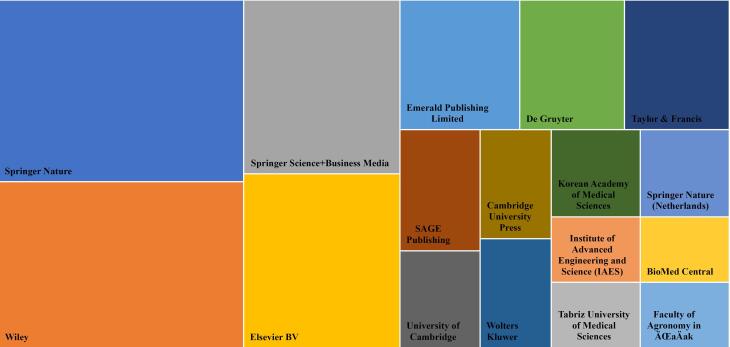
Top publishers

 As shown in Figure 5, Springer Nature, Wiley, Springer Science, Elsevier, and Emerald are the top five publishers on the topics. It highlights the importance of topics. Since most of the hijacked journals have been published by small publishers, addressing the issue of hijacked journals by standout publishers shows the concern of these publishers about the growth of hijacked journals and the dissemination of non-peer review science into the literature.

**Figure 6:**
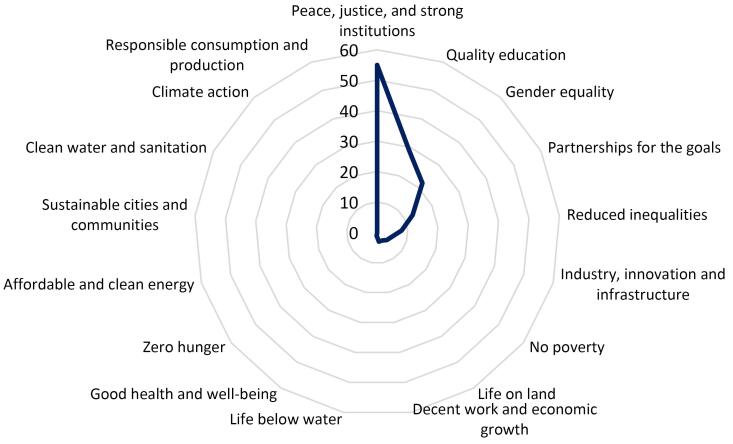
Alignment with sustainable development goals

 The published papers on the topic of hijacked journals usually contribute to sustainable development goals (SDG) provided by the United Nations.^16,17^ The top SDGs are “peace, justice, and strong institutions”, “quality education”, and “gender equality” (Figure 6). The hijacked journals publish non-peer-reviewed science and compromise the integrity of the scientific record.^1,3^ This can hurt the trust of academic institutes. Hijacked journals charge authors^3^ (and sometimes readers); This can create financial strain and exacerbate existing inequalities. This journal deceives researchers, and as their paper will be published in non-legitimate journals, the author’s scientific careers will face challenges. Hijacked journals waste authors’ efforts, time, and institutes’ budgets. These journals do not meet academic standards and negatively impact the quality of education. These journals can hurt the integrity of science in various disciplines, especially health science, with negative consequences for public health, environmental protection, and social policy.^18-20^ The published papers by hijacked journals are a warning for sustainable development; a study shows that the content published in hijacked journals can contribute to SDGs, but they are non-peer reviews so, they can disseminate misinformation or hurt institution ranks.^21^

**Figure 7:**
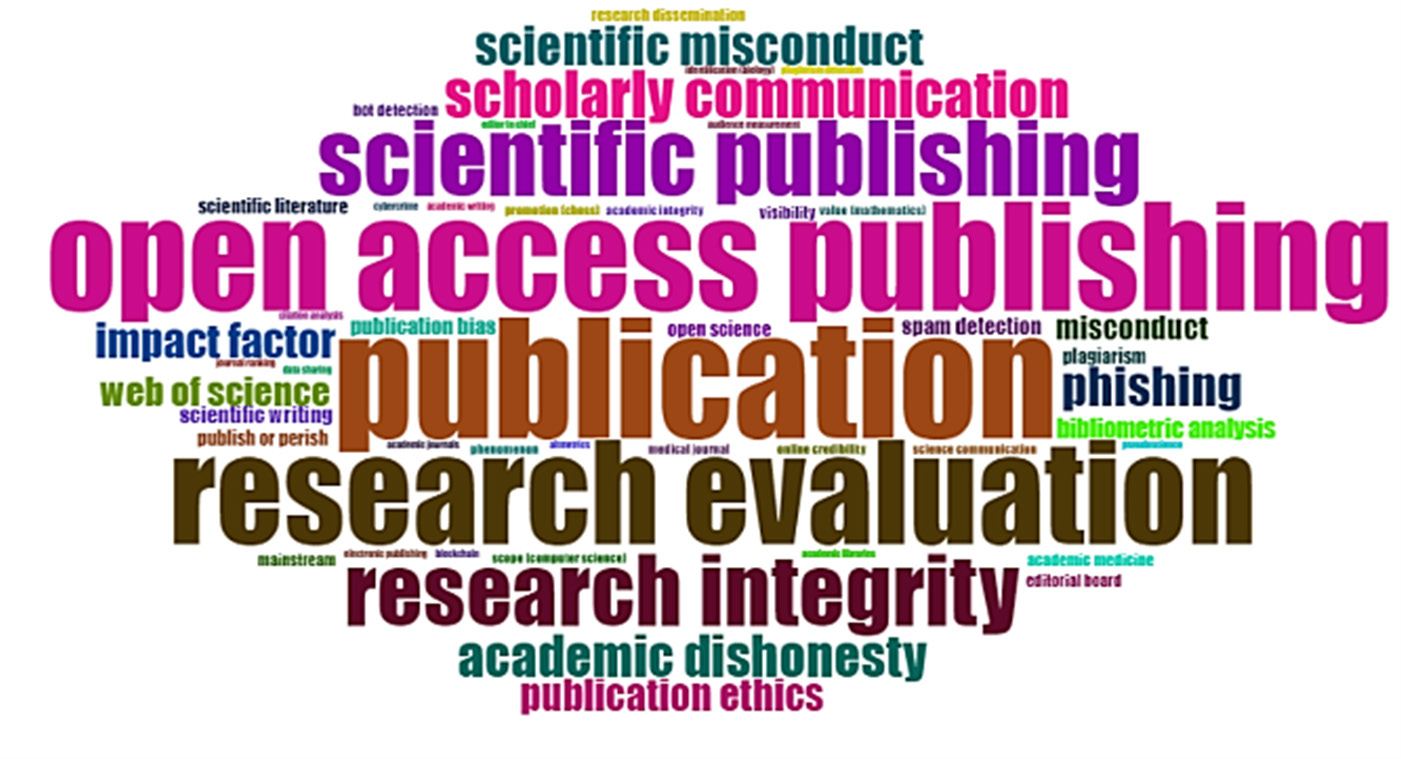
Word cloud

**Figure 8:**
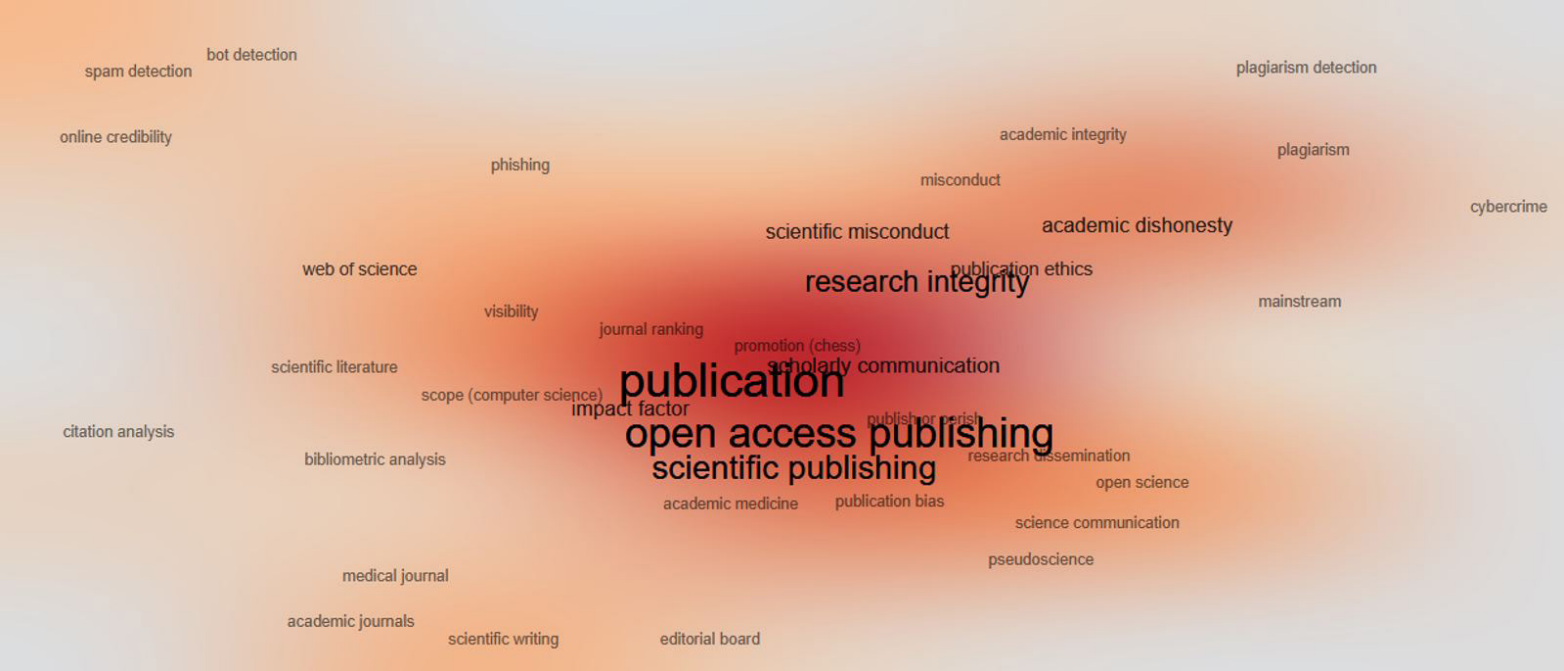
Co-occurrence Network

Figures 7 and 8 show the top frequent keywords and words co-occurrence in sequence. They somewhat provide similar concepts such as the primary topics of published papers, but there are keywords about the hijacked journals detection. The keywords bot detection, impact factor, plagiarism, spam detection, phishing, citation analysis, and similar keywords emerged due to available publications on the topic of hijacked journal detection or prevention. The spam call for papers is key for most hijacked journals to find potential victims.^22^ They are analyses of citations to hijacked journals and plagiarism or similarity analysis to find a network of hijacked journals.^23,24^ The evidence indicates that some hijacked journals use the same content as each other.^23^ The hijacked journals may use bogus impact factors to look legitimate or gain more visibility, especially among authors from developing countries.^25,26^ Some research considers hijacked journals similar to phishing attacks and presents methods for the detection of them.^15^ There are machine learning or artificial intelligence-based methods for hijacked journal detection.^5^ However, the simplest method that can be used is a list of hijacked journals. The most recent and updated one is the Retraction Watch Hijacked Journals Checker (RWHJC), which is regularly updated.^27^

**Figure 9:**
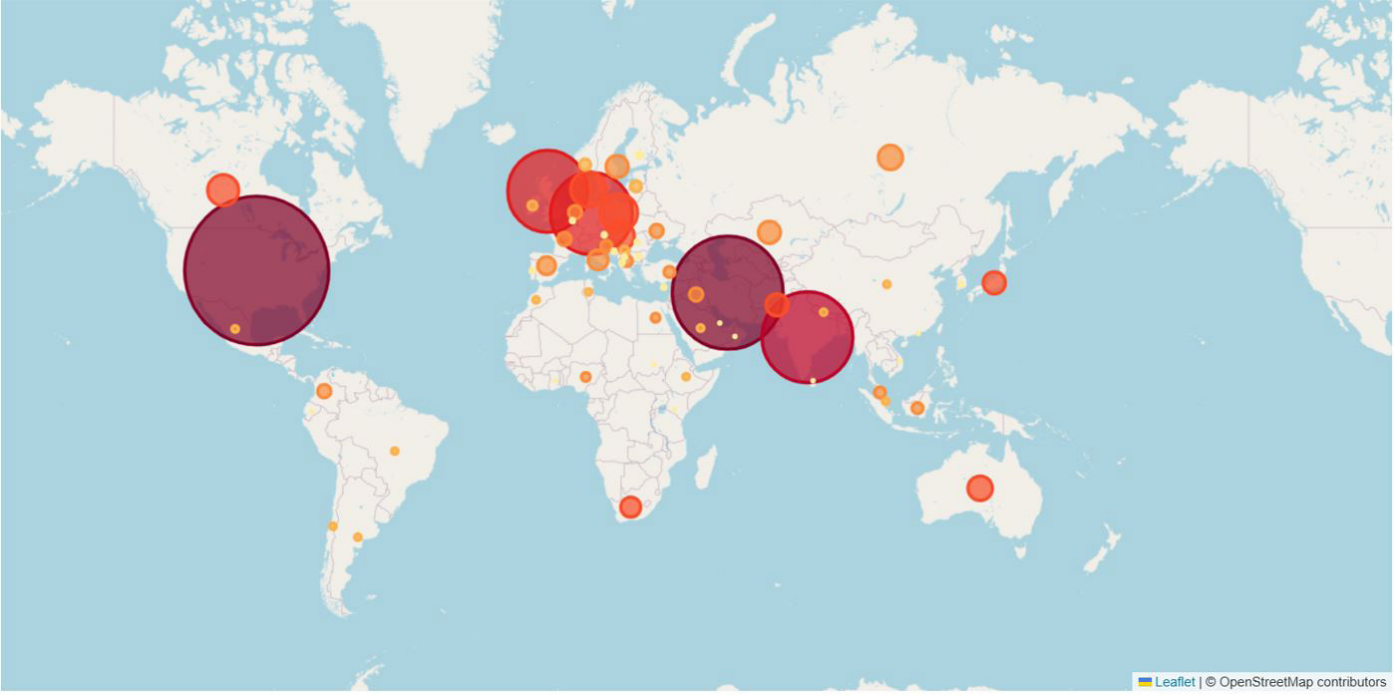
Authors’ countries (visualization made with Poe and AI, based on extracted data)

 The majority of publications about hijacked journals are from Iran, the USA, and India. A limited number of authors are dedicated to the continuous research of this topic. This means that this field is looking for continued research and funding support to grow (Figure 9).

## The dark side

 Despite considerable research on the topic, hijacked journal literature still presents certain drawbacks. The current number of hijacked journals remains unclear due to the complexity of their detection process. The growth rate of this journal is not clear, as there are hijacked journals that disappeared after launching without any detection. It’s unclear how many hijacked journals have indexed their content in citation bases, and we don’t know how many of these journals are indexed in reputable databases. The total number of citations to published manuscripts in hijacked journals is not accessible. We have not yet inspected the patent-based citations to published papers in hijacked journals. Addressing the many open questions surrounding hijacked journals is necessary.

## The future solution

**Figure 10:**
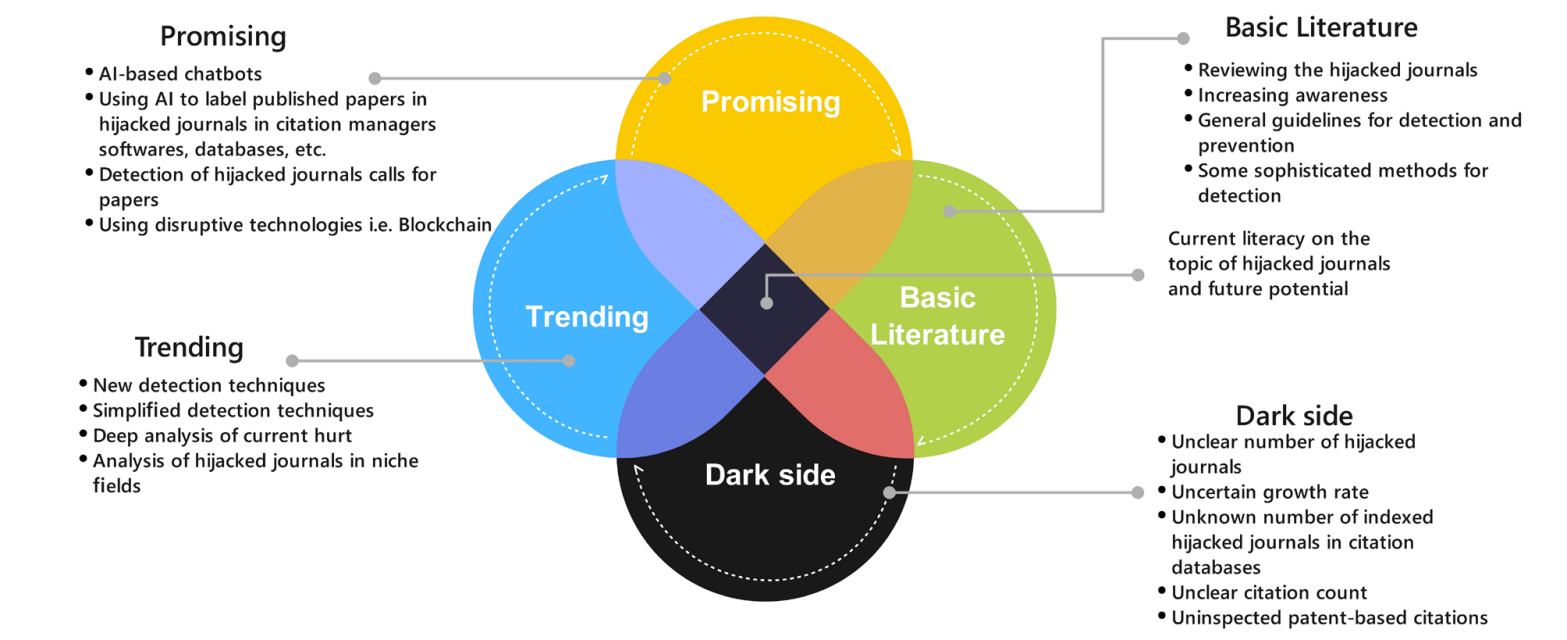
Current literacy on the topic of hijacked journals and future potential

 It seems that the use of AI for dealing with hijacked journals can be promising. AI can be used to develop interactive chatbots for users and help them query journals and analyze possible ones against some criteria to ensure a journal is hijacked or original. The anonymized transferred data from users’ accounts can be analyzed to detect potential hijacked journals. Some studies indicate that most of the victims of hijacked journals are from certain countries or even certain research institutes^4,21^; when queries about a certain journal from a geographic area increase in an AI chatbot, the potentially questionable journals can be detected and several victims will be eliminated. The AI tool can be used to find suspected calls for papers and label them as fraud. The AI tool can be developed to analyze data in citation bases and find potential indexes from the side of hijacked journals. All of these are topics for future research and request efforts, time, and budgets. Figure 10 illustrates current literacy on the topic of hijacked journals and future potential in a simple manner.

## Conclusion

 Various papers in the literature discuss hijacked journals and try to increase the awareness of authors and eliminate victims. However, there is a lack of papers to direct future research. It seems that the literature on hijacked journals is mature, but this consideration is not true. Most papers only introduce the current harm of hijacked journals or methods for detection of them, but no easily usable tool is available to all researchers. The current editorial tried to provide a close view of the current literature and provide some key trends for future research in this area.

 The majority of analyses in this edition are based on a data science approach and maybe some tolerances in value. Also, we used OpenAlex to cover the most possible extent of published contents; it means that some papers completely contribute to hijacked journal literature and this contribution for some papers may be limited.

## AI Usage

 The AI tools have been used to improve readability in some parts of the paper. The other usage of AI tools has been declared in the methodology section.

## Competing Interests

 None declared.

## Ethical Approval

 Not applicable.
